# Hypoxia mediates immune escape of pancreatic cancer cells by affecting miR-1275/AXIN2 in natural killer cells

**DOI:** 10.3389/fimmu.2023.1271603

**Published:** 2023-11-15

**Authors:** Zhenglin Ou, Yebin Lu, Dayong Xu, Zhen Luo

**Affiliations:** ^1^Department of General Surgery, Xiangya Hospital Central South University, Changsha, Hunan, China; ^2^National Clinical Research Center for Geriatric Disorders, Central South University, Changsha, Hunan, China; ^3^Department of General Surgery, The First Hospital of Changsha, Changsha, Hunan, China

**Keywords:** pancreatic cancer, hypoxia, miR-1275, Axin2, natural killer (NK) cells

## Abstract

Given the increasing incidence of pancreatic cancer and the low survival rate, the exploration of the complex tumor microenvironment and the development of novel treatment options is urgent. NK cells, known for their cytotoxic abilities and modulation of other immune cells, are vital in recognizing and killing cancer cells. However, hypoxic conditions in the tumor microenvironment have been found to impair NK cell functionality and contribute to tumor immune escape. Therefore, we aimed to uncover the mechanism through which hypoxia mediates the immune escape of pancreatic cancer cells, focusing on the influence of miR-1275/AXIN2 on NK cells. Using a combination of GEO dataset screening, Tumor Immune Estimation Resource 2.0 immunoscore screening, and the Cancer Genome Atlas data, we identified a correlation between miR-1275 and NK cells. The down-regulation of miR-1275 was associated with decreased NK cell activity and survival in patients with pancreatic cancer. Pathway analysis further linked miR-1275 expression with the hypoxic HIF1A pathway. *In vitro* experiments were conducted using the NK-92 cell, revealing that hypoxia significantly reduced miR-1275 expression and correspondingly decreased the cell-killing ability of NK cells. Upregulation of miR-1275 increased perforin, IFN-γ and TNF-α expression levels and enhanced NK cell cytotoxicity. Additionally, miR-1275 was found to bind to and inhibit AXIN2 expression, which when overexpressed, partially alleviated the promotive effect of upregulated miR-1275 on NK-92 cell killing ability. In conclusion, this research underscores the critical role of the miR-1275/AXIN2 axis in hypoxia-mediated immune escape in pancreatic cancer, thus opening new potential avenues for treatment strategies.

## Highlight

1. miR-1275 is low expressed in natural killer (NK) cells of pancreatic cancer patients.
2. Up-regulation of miR-1275 partially reversed the inhibitory effect of hypoxia on NK cell cancer cell killing.
3. AXIN2 is a downstream target gene of miR-1275.
4. Hypoxia promotes immune escape of pancreatic cancer cells by inhibiting the NK cell miR-1275/AXIN2 axis.

## Introduction

Pancreatic cancer is a commonly seen gastrointestinal malignancy with a rapidly increasing incidence in recent years (1). Early diagnosis remains very difficult for pancreatic carcinoma patients, and most pancreatic cancer patients are diagnosed at an advanced stage with an impaired outcome (2). The five-year relative survival rate for pancreatic carcinoma patients is < 8% (3). So far, surgery offers the only chance for curative treatment. Nevertheless, most patients are unresectable due to delayed disease presentation (4). Therefore, to improve the pessimistic prognosis for patients with pancreatic cancer, novel treatment options and a better knowledge of the intricate matrix crosstalks in the tumor microenvironment seem to be urgently required.

Natural killer (NK) cells have been recognized as a critical innate immune system member with direct natural cytotoxicity, antibody-dependent cytotoxicity, and indirect modulation of the secretion of other immune cell functions with inflammatory cytokines and chemokines (5, 6). NK cells have been found to recognize ligands expressed on cancer cell surface surfaces, thereby killing cancer cells (7). However, impaired NK cell-related cytotoxicity has been reported to result in tumor immune escape (8). For example, Nk cell dysfunction is significantly linked to immune escape from pancreatic carcinoma cells (9).

Hypoxia contributes to the immune escape of several tumors (10). Hypoxia is one of the factors contributing to impair NK cytotoxic activity within the tumor microenvironment (TME). Hypoxic TEM has been found to impair NK cell function through inhibiting signals including NKG2D, NKp30 and CD16 (11). The hypoxic TME can not only directly regulate intracellular signaling, but also destroy functional molecules released by NK cells, such as granzyme B (12). Hypoxia has been previously reported to cause immune escape from pancreatic cancer by inhibiting the expression levels of NK cell activation receptors MICA and MICB within tumor cells (13). In addition, the hypoxic TME results in NK cells’ failure to survive or proliferate thereby causing immune escape from pancreatic cancer (14). Therefore, exploring the mechanism of the dysfunction of NK cells due to hypoxia is crucial for the treatment of immune escape in pancreatic cancer.

MicroRNAs are a group of small noncoding RNAs, which exert a negative regulatory effect on gene expression through mRNA destabilization or translation repression. miRNAs have been revealed to exert crucial effects on a number of biological processes, such as cellular growth, apoptosis, cellular differentiation, immune response, and tumor development (15–17). Moreover, miRNAs play a vital role in the regulation of NK cell function. Previous reports have shown that extracellular vesicle-derived miR-150-5p promotes hypoxic pulmonary cancer development via altering NKC phenotype and impairing NKC function (18). miR-146a is able to suppress the function of NK cells via binding to STAT1 (19). However, miRNA regulation of NK cell function within pancreatic carcinoma remains unclear.

Herein, miR-1275, a differentially expressed miRNA associated with NK cell scoring, was obtained by screening the GEO dataset, and miR-1275 expression and its association with NKCs in pancreatic cancer tissues were validated. Pathway enrichment analysis demonstrated the association between miR-1275 and hypoxia pathway. After induction of NK cells by hypoxia, we determined the NK cell-mediated cancer cell killing ability and investigated the molecular mechanism by which the miR-1275/AXIN2 axis in NK cells inhibits its killing ability against pancreatic cancer cells.

## Methods

### GSE85589 variance analysis

The microarray GSE85589 was obtained from Gene Expression Omnibus (GEO, https://www.ncbi.nlm.nih.gov/geo/). The R language limma package was employed to perform differential gene analysis on this dataset, setting the screening parameters to |logFC| > 0.4 and adjusted P.val < 0.05. Data were collected from serums from 88 subjects with pancreatic carcinoma, 101 subjects with intrahepatic cholangiocarcinoma (ICC), 7 subjects with stomach carcinoma (SC), 5 subjects with colorectal carcinoma (CC), 2 subjects with gastrointestinal stromal tumor (GIST), 10 subjects with cholelithiasis. Serum samples were taken from 19 subjects who were clinically classified as healthy at the time of participation. We only compared the serum samples miRNA differences between 88 pancreatic cancer patients and 19 normal control patients.

### Correlation of differential miRNAs and immune cell types

Immune cell scoring was performed by immune scoring TIMER2.0 (http://timer.cistrome.org/) on 163 TCGA-PAAD pancreatic cancer tissue expression microarrays using two algorithms (quanTIseq and MCPCounter) (20, 21) including characteristic parameters of NK (Natural Killer) cells. The 82 differentially identified miRNAs were crossed by 2 sets of algorithms to obtain the most closely associated miRNAs with NK (*r >* 0.15 or *<* -0.15, *p.*value < 0.05).

### Clinical sample

The study protocol of using clinical samples for research was approved by the Medical Ethics Committee of Xiangya Hospital, Central South University. 24 paired PC and non-tumor para-carcinoma tissue specimens were obtained from subjects that were surgically removed from our hospital, as well as blood samples from patients. In addition, Blood samples from the same number of healthy individuals who visited our hospital for physical examination were harvested as controls. All tissues were formalin-fixed or stored within a -80°C fridge. Written informed consent was obtained from all patients enrolled.

### H&E staining

After being dehydrated and paraffin-embedded, the pancreatic cancer tissues were sectioned at a thickness of 5 μm, dewaxed with xylene (Sigma-Aldrich, St. Louis, MO, USA), re-hydrated in graded ethanol, subjected to 5-min staining with hematoxylin (Sigma-Aldrich), and then fractionated for 30 s using hydrochloric acid alcohol. After being immersed in tap water for 15 min, paraffin sections were subjected to 3-min staining with eosin (Sigma-Aldrich), and then dehydrated in graded alcohol with xylene transparency. A microscope was employed to observe sections sealed with neutral resin.

### Immunofluorescent staining and fluorescence *in situ* hybridization

Paraffin sections of pancreatic carcinoma tissue samples were routinely dewaxed and rehydrated, followed by a 10-min incubation with 3% H_2_O_2_ and then a 10-min incubation with sodium citrate solution 0.01 M, pH 6.0 at 95°C. Then, slices were closed with 5% goat serum for 20 min at room temperature (RT), followed by an overnight incubation at 4°C with anti-CD16 (1:100; bs-6028R, Bioss, Woburn, MA, USA). Alexa flour 647-labeled secondary antibody (1:200; ab150079; Abcam, Cambridge, MA, USA) was then added and the nuclei were then subjected to DAPI staining. A fluorescent microscope was utilized to observe slices.

For miR-1275 co-localized with CD16, slices were dewaxed, proteinase K digested and antigen repaired, and then salmonid sperm DNA with hybridization buffer was diluted and subjected to a 60-min incubation at 37° to pre-hybridize slices. 1 μM hybridization buffer was employed to dilute probe miR-1275 conjugated with DIG (5’- GACAGCCUCUCCCCCAC-3’), and slices were subjected to an overnight hybridization within a constant temperature incubator at 40°C. Sections were rinsed using SSC liquid with a gradient concentration, followed by a 30-min incubation at RT using 10% rabbit serum, and a 50-min incubation at 37°C using anti-DIG-HRP antibody and then a 5-min incubation at RT away from light using FITC-TSA. Next, sections were subjected to a 30-min incubation at RT using BSA and then an overnight incubation at 4°C with CD16 antibody (bs-6028R, Bioss) prepared in PBS (1:200 dilution) in a wet box. Following the addition of a secondary antibody (Cy3-labeled), sections were incubated for 50 min at RT. Finally, the nuclei were subjected to DAPI staining, an anti-fluorescence quenching sealer was employed to seal section and a fluorescence microscope (Olympus, Tokyo, Japan) was applied to observe sections. CD16 expression is shown by red fluorescence and miR-1275 expression is shown by green fluorescence.

### Cell culture

The NK cell line NK-92 cells and the pancreatic carcinoma cell line Panc-1 cells were procured from the American Typical Culture Collection (ATCC). Panc-1 cell line was cultivated in dulbecco’s modified eagle medium (DMEM) containing 10% fetal bovine serum (FBS), and 0.1% penicillin-streptomycin. NK-92 cell line was cultivated in RPMI-1640 media containing 20% FBS and 150 IU/mL recombinant IL-2. The cells were cultured in a humidified environment at 37°C with 5% CO_2_. Primary natural killer (NK) cells were isolated from peripheral blood mononuclear cells (PBMCs) of healthy volunteers and pancreatic cancer patients using Magnetic cell sorting (StemCell Technologies, Vancouver, BC, Canada), which were then used for RNA or protein isolation.

### Cell transfection

Overexpression plasmids (AXIN2 oe) and blank vector (pcDNA-3.1) were procured from YouBio (Changsha, China). The miR-1275 agonist agomir-1275 and the antagonist antagomir-1275 and their negative controls agomir-NC and antagomir-NC were procured from Genepharma (Shanghai, China). An electromicroporator system (Bio-rad, Hercules, CA, USA) was employed as per the manufacturer’s protocol to perform transfections. The primers are listed in **Table S1**.

### Hypoxia treatment

Untransfected or transfected NK-92 cell line was inoculated to six-well plates, followed by a 24-h incubation in conditions containing 15% O_2_, 10% O_2_, 5% O_2_ and 1% O_2_, then cells were harvested for further investigation. Besides, HIF1α inhibitor (Methyl 3-[[2-[4-(2-adamantyl) phenoxy] acetyl] amino]-4-hydroxybenzoate, CAY10585) (HY-13671, MedChemExpress, Shanghai, China) were treated to NK-92 cells at concentrations of 20 µM for 24 h (22).

### qRT-PCR

TRIzol reagent (Invitrogen, Carlsbad, CA, USA) was employed as per the manufacturer’s protocol to extract total RNA from tissues or NK cells. FastKing cDNA synthesis kit (TIANGEN, Beijing, China) was utilized to reversely transcribe RNA to cDNA. SuperReal PreMix Plus (SYBR Green) (TIANGEN) was applied to perform qRT-PCR upon the Fast Real-Time PCR 7500 System. U6 was utilized as miRNA internal control and GAPDH as mRNA internal control. The 2-ΔΔCt method, ΔΔCt = experimental group (Ct target gene-Ct internal reference) - control group (Ct target gene-Ct internal reference), was employed to analyze data. The primers utilized in this study are as follows **Table S2**.

### Enzyme linked immunosorbent assay

After cell transfection and hypoxia treatment, NK-92 cells were followed by co-cultured with Panc-1 cells ratios of 25:1 for 6 hours at cell culture incubator. Next, the cell culture supernatants were harvested for ELISA analysis. The ELISA kits were utilized to measure perforin, IFN-γ and TNF-α levels within NK-92 cell culture supernatants. Then a microreader was applied to measure the absorbance at 450 nm. The perforin (CSB-E09313h, CUSABIO, Wuhan, China), IFN-γ (PI521, Beyotime, Shanghai, China) and TNF-α (PT518, Beyotime) ELISA kits were performed according to the manufacture’s instruction.

### Cell killing assay

NK-92 cells were transfected, followed by a 24-h incubation in normoxic/hypoxic conditions and a 6-h incubation with Panc-1 cells at a ratio of 6.25:1, 12.5:1, 25:1, and 50:1. The supernatant was discarded, followed by the addition of 20 μL MTT (5 mg/mL; Sigma-Aldrich) and a 4-h incubation. Then, after discarding the supernatant, methan was dissolved in 200 μL of DMSO. Optical Density (OD) values at 490 nm were assessed. Panc-1 cell survival in normoxic environments was defined as 100%.

### Western blot

RIPA buffer (Beyotime) was employed to extract total protein and lyse cells, and BCA kit (Beyotime) was employed to detect the protein content. Following electrophoresis by SDS-PAGE (sodium dodecyl sulfate-polyacrylamide gel electrophoresis), the separated proteins were electroblotted from the gel onto PVDF membranes and closed using skim milk. The isolated proteins were then subjected to an overnight incubation at 4°C using anti-HIF1A (1:1000; ab179483; Abcam), anti-AXIN2 (1:1000; ab109307; Abcam) and anti-GAPDH (1:2500; ab9485; Abcam). Goat anti-rabbit IgG (1:2000; ab6721; Abcam) coupled with horseradish peroxidase was then used, followed by a 2-h incubation at RT. GAPDH was utilized as an internal control. An enhanced chemiluminescence substrate kit (Beyotime) was employed to visualize the proteins.

### Dual luciferase reporter gene assay

To investigate the binding relationship of miR-1275 to AXIN2, AXIN2 3’UTR wild-type sequence and mutant sequence were cloned into psichech2 luciferase reporter gene to construct AXIN2 3’UTR-WT and AXIN2 3’UTR-MUT plasmids. The reporter plasmids and agomir-NC, agomir-1275, antagomir NC and antagomir-1275 were cotransfected into 293t cells with Lipofectamine 2000. A dual luciferase assay system (Beyotime) was employed to measure luciferase activity, and the sea kidney luciferase was utilized as an internal reference.

### Statistical analysis

All experimental data were expressed in terms of the mean ± standard deviation (SD). GraphPad Prism 8.0 software was applied to perform student’s t-test, one-way ANOVA followed Tukey *post hoc* test or Dunnett’s Test. The significance level was set at *P*<0.05.

## Result

### Screening and analysis of miRNA in blood samples from pancreatic cancer patients

**Figure 1:**
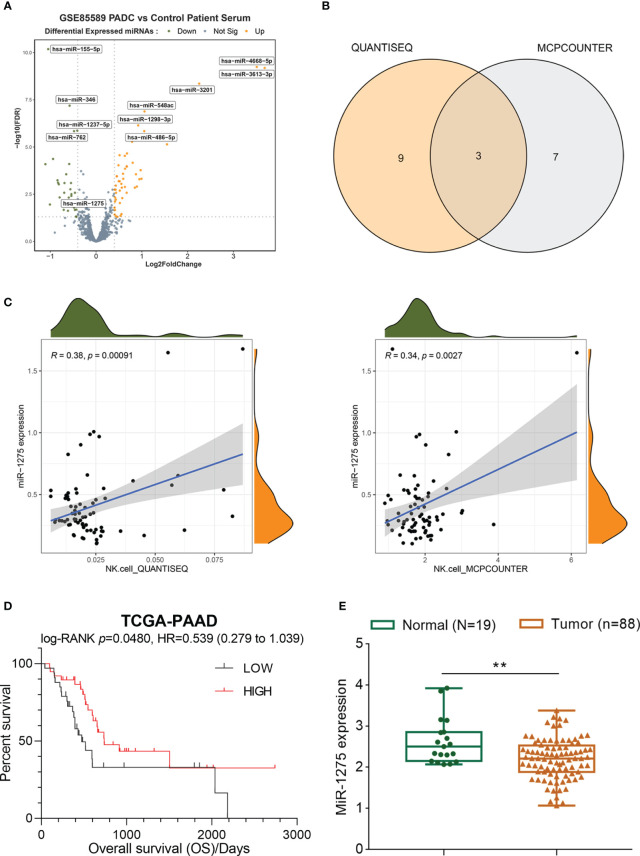
Screening and analysis of miRNA in serum samples from pancreatic cancer patients. **(A)** Volcano map of differentially expressed miRNAs in pancreatic cancer based on the microarray dataset GSE85589. **(B)** Crossover results of immune scoring TIMER 2.0 for 82 differential miRNAs with 2 sets of algorithms. **(C)** NK cell scoring of miR-1275 by quanTIseq and MCPCounter. **(D)** Survival analysis of miR-1275 in pancreatic cancer data from TCGA-PAAD. **(E)** Expression of miR-1275 in GSE85589. ** P < 0.01.

GSE85589 was downloaded from GEO and analyzed to compare differential miRNAs within serum specimens from patients with pancreatic carcinoma and healthy control patients, resulting in 32 down-regulated miRNAs and 50 up-regulated miRNAs (**Figure 1A**). Immunocellular scoring of 82 differential miRNAs was performed by immune scoring TIMER 2.0. 3 miRNAs (hsa-miR-628-5p, hsa-miR-155-5p, hsa-miR-1275) associated most closely with NK were obtained by crossover results of two algorithms (QUANTISEQ and MAPCounter) (**Figure 1B**), where miR-1275 was positively associated with NK cell score in pancreatic cancer in both algorithms (**Figure 1C**). Survival analysis of miR-1275 in TCGA-PAAD pancreatic cancer data showed that miR-1275 acted as a protective factor for pancreatic cancer patients’ overall survival (HR=0.539, 95% CI =0.279-1.039, p.value=0.048) (**Figure 1D**). The results of GSE85589 data analysis demonstrated that miR-1275 was lowly expressed within serum samples from pancreatic cancer patients (**Figure 1E**). Therefore, we think that miR-1275 may be an NK-related oncogenic miRNA in pancreatic cancer.

### miR-1275 is lowly expressed in pancreatic cancer tumors

**Figure 2:**
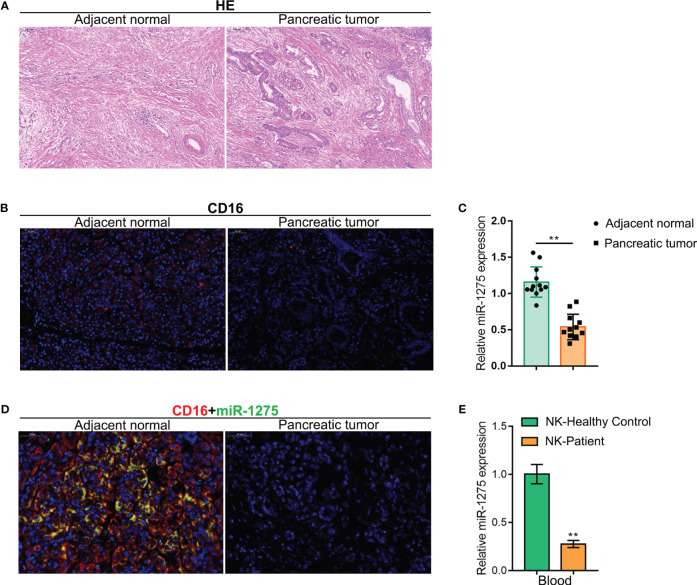
miR-1275 is Lowly Expressed in Pancreatic Cancer Tumors. **(A)** H&E staining for pancreatic cancer tissue and adjacent normal tissue. **(B)** IF assays for expression levels of the NK cell marker CD16 in tissues. **(C)** qRT-PCR to detect miR-1275 expression within pancreatic carcinoma and adjacent normal tissues. **(D)** IF detection of CD16 co-localization with miR-1275 and changes in expression of CD16 with miR-1275. **(E)** qRT-PCR to detect changes in miR-1275 expression in NK cells from healthy volunteers and pancreatic cancer patients. **P < 0.01.

To further confirm miR-1275 expression within pancreatic carcinoma patients, the pancreatic carcinoma tissue samples and adjacent normal tissue samples were collected from pancreatic cancer patients and blood specimens from patients with pancreatic cancer and healthy control. Tissues were subjected to H&E staining (**Figure 2A**). We further observed the level of NK cell marker CD16 in pancreatic cancer tissues by IF, and NK cell infiltration showed to be reduced within pancreatic carcinoma tissue samples than adjacent normal samples (**Figure 2B**). miR-1275 expression within pancreatic carcinoma tissue samples showed to be dramatically decreased compared with para-carcinoma tissue samples, as shown by qRT-PCR in tissue samples (**Figure 2C**). To demonstrate the association of miR-1275 with NK cells, we used a fluorescent probe of miR-1275 to co-stain cells with CD16 immunofluorescence. The results showed that CD16 fluorescence colocated with miR-1275 fluorescence, and both CD16 fluorescence and miR-1275 fluorescence were reduced in pancreatic cancer tissues (**Figure 2D**). We further collected NKs in the peripheral blood from healthy volunteers and pancreatic cancer patients by magnetic bead sorting, and conducted qRT-PCR to detect miR-1275 expression in NK cells. miR-1275 levels within NKs were shown to be dramatically reduced within patients (**Figure 2E**). Taken together, miR-1275 is lowly expressed within pancreatic carcinoma tissues and a reduced expression level of miR-1275 is linked to a possible reduction in NK cell infiltration.

### miR-1275 expression-related genes pathway enrichment analysis

**Figure 3:**
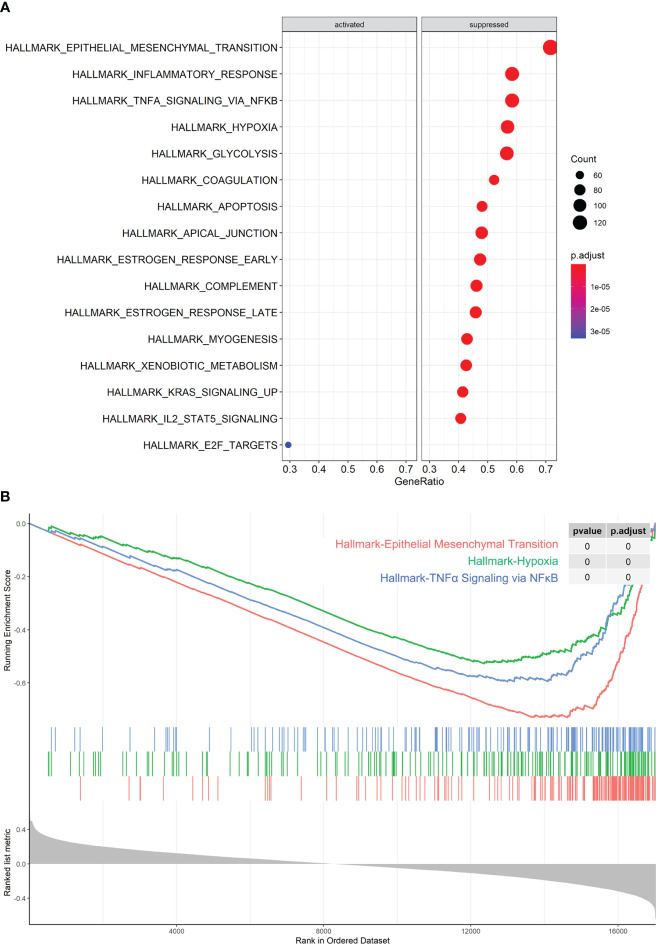
miR-1275-Related Pathway Enrichment Analysis. **(A, B)** GESA analysis of pathway enrichment of miR-1275 in TCGA-LUAD data.

The miR-1275 expression correlated genes were collected from TCGA-LUAD and association enrichment analysis were performed by GESA. As the results shown, those genes were negative associated with hypoxic pathways, epithelial-mesenchymal transition (EMT), and inflammatory response and factor pathways such as NF-KB, while it was also associated with various immune cell signaling pathways such as IL2-STAT5 (**Figures 3A, B**).

### Hypoxia reduced miR-1275 expression in NK cells and reduced the cytotoxicity of NK cells

**Figure 4:**
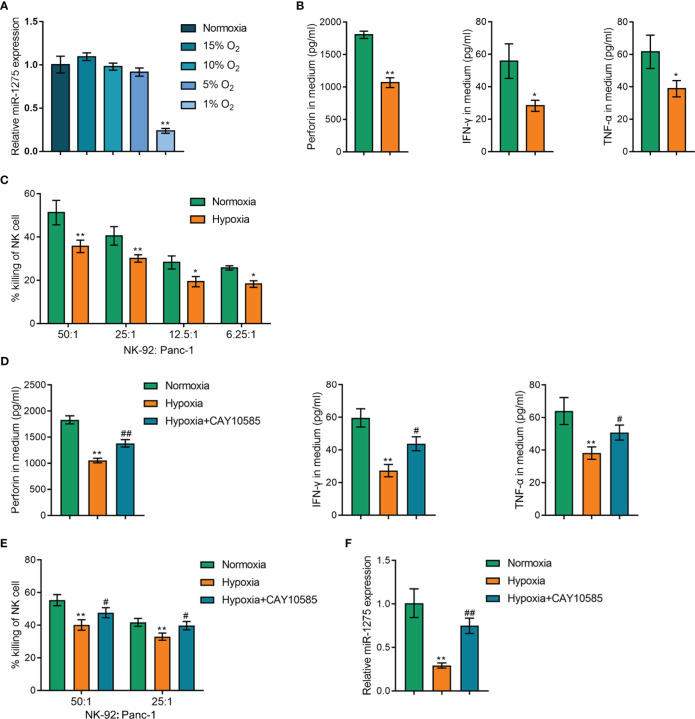
Hypoxia Affects Cancer Cell Killing Ability of NK cells. **(A)** qRT-PCR assay of miR-1275 expression levels within NK-92 cells treated with different oxygen concentrations (15% O_2_, 10% O_2_, 5% O_2_ and 1% O_2_). **(B)** NK-92 cells were cultivated under hypoxia and normoxia for 24 h and the supernatants were collected to detect the levels of the relevant immune factors’ perforin, IFN-γ and TNF-α by ELISA assay. **(C)** NK-92 cells were incubated with Panc-1 (ratio is 50:1, 25:1, 12.5:1, 6.25:1) for 6 h after hypoxia or normoxia stimulation and the killing of Panc-1 cells by NK-92 cells was determined by MTT assay. **(D)** NK-92 cells were cultivated under hypoxia or normoxia for 24 h, and then treated with 20µM HIF1α inhibitor CAY10585 for 24 h and the supernatants were collected for detecting the levels of the relevant immune factors’ perforin, IFN-γ and TNF-α by ELISA assay. **(E)** NK-92 cells were incubated with Panc-1 (ratio is 50:1, 25:1) for 6 h after hypoxia or normoxia stimulation and CAY10585 treatment, and the killing of Panc-1 cells by NK-92 cells was determined by MTT assay. **(F)** NK-92 cells were cultivated under hypoxia or normoxia and CAY10585 for 24 h and the miR-1275 expression was detected by RT-qPCR. *P < 0.05, **P < 0.01, in comparison with Normoxia, *# P < 0.05*, *##P < 0.01*, in comparison with Hypoxia.

As previously reported, tumor-infiltrating NK cell activity could be raised by HIF-1α inhibition (23), suggesting that hypoxia may act directly on NK cells to modulate their immune activity. And miR-1275 correlated genes were negative associated with hypoxic pathways. Therefore, we speculate that hypoxia affects miR-1275 to regulate NK cell activity in pancreatic cancer. Next, NK-92 cells were cultivated in different hypoxic environments and qRT-PCR was conducted to detect miR-1275 expression in the cells, and miR-1275 expression within NK cells showed to be significantly reduced when the oxygen content was 1% (**Figure 4A**). Therefore, subsequent experiments were performed under 1% oxygen conditions. The levels of relevant immune factors secreted by NK-92 cells under hypoxic conditions and Panc-1 incubation were measured by ELISA, and hypoxic conditions showed to considerably inhibit perforin, IFN-γ and TNF-α expression levels within NK-92 cells (**Figure 4B**). Normoxia- and hypoxia-treated NK-92 cells were co-cultured with pancreatic cancer Panc-1 cells and Panc-1 cell viability was examined, and the results showed that the capacity of hypoxia-treated NK-92 cells to kill cancer cells was significantly reduced compared with that of NK-92 cells under normoxia (**Figure 4C**). To inhibit HIF-1α activity, 20µM HIF1α inhibitor CAY10585 was used in NK-92 cells for 24 h. As **Figure 4D** showed, CAY10585 notably reversed the effects of hypoxia on immune factors by promoting immune factors levels in NK-92 cells under hypoxic conditions. The capacity of hypoxia-treated NK-92 cells to kill cancer cells was markedly increased by CAY10585 treatment (**Figure 4E**). Moreover, miR-1275 expression within NK cells under hypoxic conditions was observably promoted by CAY10585 treatment (**Figure 4F**). All these results show that hypoxic conditions can inhibit the capacity of NK cells to kill cancer cells.

**Figure 5:**
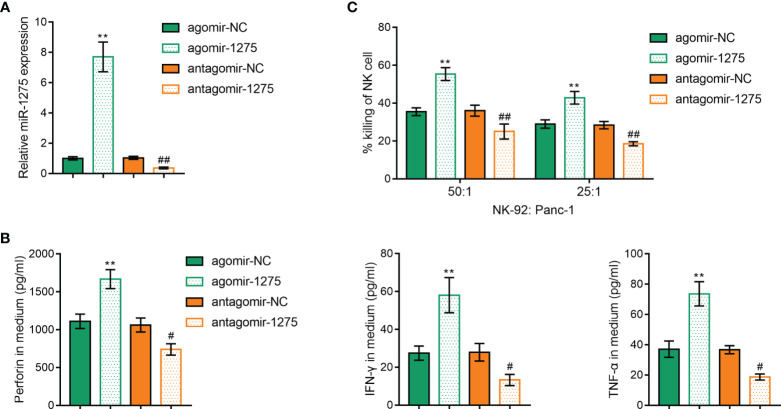
Hypoxia Affects miR-1275’s Regulation of Cancer Cell Killing Ability of NK cells. **(A)** RT-qPCR was applied to detect miR-1275 levels in NK-92 cellsafter agomir-1275 and antagomir-1275 transfection. **(B, C)** ELISA assay was used to detect perforin, IFN-γ and TNF-α levels **(B)** and MTT assay was used to detect killing of Panc-1 cells by NK-92 cells **(C)** after NK cell transfection under hypoxic conditions. **P < 0.01, in comparison with agomir-NC, *# P < 0.05*, *##P < 0.01*, in comparison with antagomir-NC.

Moreover, the roles of miR-1275 on NK cell activity in pancreatic cancer under hypoxic conditions were investigated. The function of miR-1275 was next examined by upregulating and inhibiting miR-1275 expression by agomir-1275 and antagomir-1275, and verified by qRT-PCR (**Figure 5A**), which showed that upregulation of miR-1275 under hypoxic conditions and Panc-1 incubation significantly increased the expression levels of perforin, IFN-γ and TNF-α (**Figure 5B**) and increased the cancer cell killing capacity of NK-92 cells (**Figure 5C**). The above results suggest that hypoxia inhibits the cancer cell killing ability of NK cells through suppressing miR-1275 expression.

### miR-1275 targets binding to AXIN2 3’UTR and inhibits its expression

**Figure 6:**
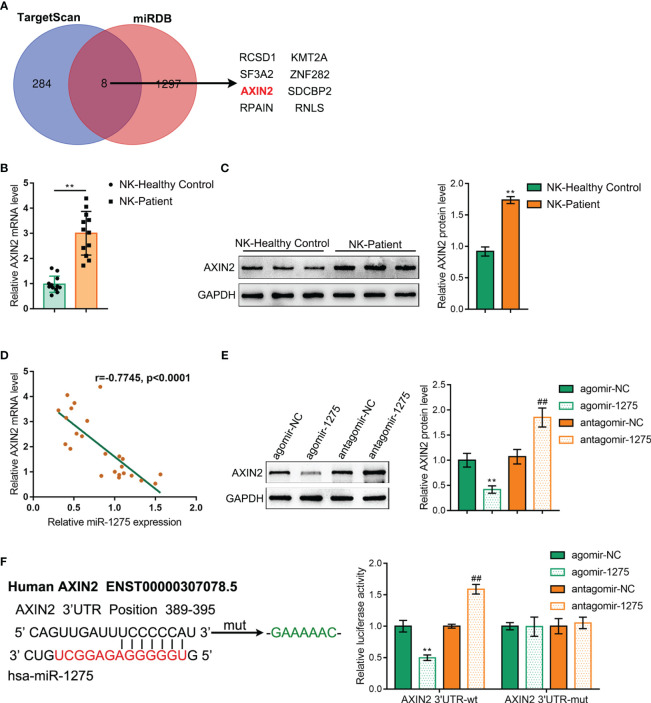
miR-1275 targets binding to AXIN2 3’UTR and inhibits its Expression. **(A)** Venn diagram of miR-1275 downstream target genes predicted via TargetScan and miRDB online database. **(B)** qRT-PCR and **(C)** Western blot detection of the expression level of AXIN2 within blood NKs isolated from the healthy volunteers and patients with pancreatic carcinoma. In comparison with the healthy control group, **P < 0.01. **(D)** Pearson correlation analysis of AXIN2 mRNA with miR-1275 in isolated NK cells. **(E)** Western blot detection of AXIN2 protein contents in NK-92 cells. **(F)** Dual luciferase reporter gene validation of miR-1275 binding to AXIN2 3’UTR sequence. In comparison with agomir-NC group, **P<0.01. In comparison with antagomir-NC group, *## P<0.01*.

Next, we explored the downstream mechanism of miR-1275. miR-1275 downstream target genes were predicted via TargetScan and miRDB online database and screened via Venn diagram to obtain eight intersecting genes (**Figure 6A**). Among them, AXIN2 was previously shown to be highly expressed in a variety of tumors (24, 25), including pancreatic cancer (26), and plays a tumor-promoting role. However, the functions of AXIN2 on NK cells under hypoxic conditions remains vague. Then, RT-PCR and Western blot were carried out to detect AXIN2 expression level within NK cells sorted from healthy volunteers and pancreatic cancer patient’s blood samples, and AXIN2 expression within NK cells from pancreatic carcinoma patients showed to be dramatically increased compared with healthy control (**Figures 6B, C**). Pearson correlation analysis was conducted to analyze the correlation between the mRNA level of AXIN2 and miR-1275 within NK cells, and miR-1275 was shown to be negatively linked to AXIN2 mRNA expression (**Figure 6D**). Western blot was performed to detect AXIN2 expression level within NK-92 cells. miR-1275 overexpression showed to considerably inhibit AXIN2 protein level, and miR-1275 inhibition was able to promote AXIN2 protein content (**Figure 6E**). As shown by the dual luciferase reporter gene assay, increased miR-1275 expression could reduce AXIN2 3’UTR wild type luciferase activity, and miR-1275 inhibition elevated AXIN2 3’UTR luciferase activity, while AXIN2 3’UTR mutant type luciferase activity showed no changes with miR-1275 level (**Figure 6F**). Taken together, miR-1275 could bind to and suppress the expression level of AXIN2.

### miR-1275 promotes NK-92 cytotoxicity under hypoxic conditions by targeting and regulating AXIN2 expression

**Figure 7:**
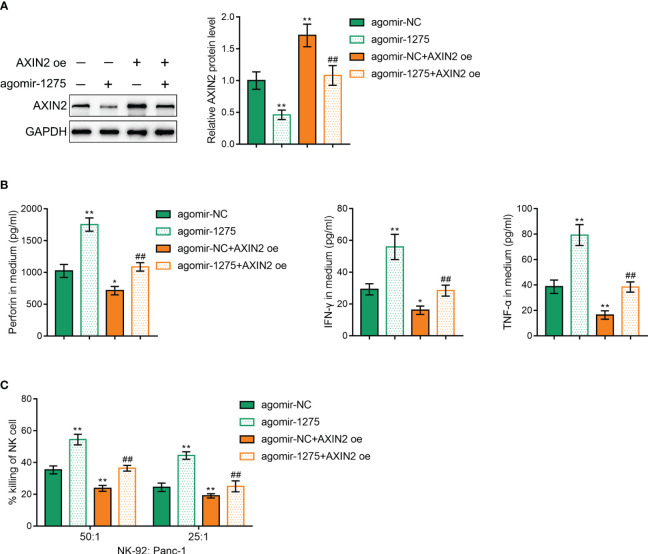
miR-1275 promotes NK-92 cell cytotoxicity under hypoxic conditions by targeting and regulating AXIN2 expression. **(A)** Western blot detection of AXIN2 protein contents within NK-92 cells after transfection. **(B)** After incubation with Panc-1 cells for 6 h, ELISA detected levels of the relevant immune factors perforin, IFN-γ and TNF-α secreted by hypoxia-stimulated NK-92 cells. **(C)** MTT detection of NK-92 cell cytotoxic effect on Panc-1 cells. In comparison with the agomir-NC group, *P < 0.05, **P < 0.01. In comparison with the agomir-1275 group, *##P < 0.01*.

Finally, we validated that miR-1275 regulates cancer cell killing ability by NK-92 cells through regulating AXIN2 expression. AXIN2 overexpression vector was cotransfected with miR-1275 agonist into NK-92 cells and its transfection efficiency was verified by Western blot (**Figure 7A**). AXIN2 overexpression under hypoxic conditions was shown to exhibit an opposite effect to miR-1275 overexpression, i.e. AXIN2 overexpression was able to inhibit perforin, IFN-γ and TNF-α secreted by NK-92 cells after incubated with Panc-1 (**Figure 7B**) and inhibited the ability of NK-92 cells to kill cancer cells (**Figure 7C**). And AXIN2 upregulation could significantly reverse the promotion of miR-1275 overexpression on NK-92 cancer cell killing (**Figures 7B, C**). The above results show that miR-1275 promotes cancer cell killing in NK-92 cells under hypoxic conditions by inhibiting AXIN2 expression.

## Discussion

MicroRNA (miRNA) are short noncoding RNA transcripts of about 18 - 25 nucleotides (27). Nowadays, mounting evidence has indicated that miRNAs are involved in NK cell function to regulate cancer immune escape, e.g. miR-544 attenuates NK cell-mediated cytotoxicity and thus promotes immune escape in hepatocellular carcinoma cells by downregulating IFN-γ (28). miR-218-5p overexpression within NK cells attenuates NK cells cytotoxicity and thus promotes immune escape from lung adenocarcinoma (29). However, there are fewer reports about miRNA regulation of NK cell function mediating immune escape from pancreatic cancer. In this study, we obtained miR-1275, a differential miRNA associated with NK cell scoring from healthy volunteers and pancreatic cancer patients via bioinformatics analysis and experimental validation., The present study demonstrated the mechanism by which hypoxia inhibited miR-1275 expression in NK cells and promoted AXIN2 expression, which in turn inhibited cancer cell killing ability of NK cells.

First, we obtained 32 down-regulated miRNAs and 50 miRNAs in pancreatic cancer by screening the public GEO dataset GSE85589. Three miRNAs associated with NK cell scoring - miR-628-5p, hsa-miR-155-5p and miR-1275 - were obtained by screening from 82 differential miRNAs by immune scoring TIMER2.0. Among them, miR-1275 was positively correlated with pancreatic cancer NK cell score in both quanTIseq and MCPCounter algorithms. Therefore, we selected miR-1275 for further study. Survival analysis of pancreatic cancer data in TCGA-PAAD revealed that miR-1275 is a protective factor for pancreatic cancer. A review of the literature revealed that miR-1275 is lowly expressed in cancers such as nasopharyngeal carcinoma (30), hepatocellular carcinoma (31), esophageal cancer (32), and breast cancer (33). However, miR-1275 within pancreatic carcinoma remains unclear. Based on both the GSE85589 dataset and pancreatic cancer clinical samples, miR-1275 was decreased within pancreatic carcinoma. Moreover, our results showed that miR-1275 overlapped with the fluorescent marker of NK cell maker CD16 and that NK cell infiltration showed to be decreased within pancreatic carcinoma tissue samples. miR-1275 expression from the peripheral blood NK cells of patients with pancreatic cancer was reduced compared with healthy control. Taken together, miR-1275 shows a low expression level within pancreatic carcinoma-associated NK cells.

miR-1275 function was investigated after determining the relevance between miR-1275 and NK cells. Pathway enrichment analysis demonstrated the negative correlation between miR-1275 and the hypoxic HIF1A pathway. The characteristic tumor microenvironment of solid tumors is hypoxia (18). It was previously reported that the hypoxic tumor microenvironment impairs the maturation and NK cells activity (34) and that HIF1A deficiency in tumor-infiltrating NK cells enhances their tumor-suppressive effects (23). Therefore, we speculate that miR-1275 might contribute to regulating the function of NK cells by the hypoxic microenvironment of pancreatic cancer. We performed hypoxia induction in NK-92 cells and revealed that the hypoxic environment significantly suppressed the killing ability of NK cells against cancer cells. Upregulation of miR-1275 expression within NK-92 cells restored the cancer cell killing ability of NK cells, while inhibition of miR-1275 expression promoted NK cell function inhibition by the hypoxic environment, indicating that miR-1275 contributes to the hypoxic regulation of NK cell function.

AXIN2 exerts a negative regulatory effect on the Wnt/β-catenin pathway which modulates a variety of cellular activities, such as the capacity of cells to proliferate, differentiate and migrate, as well as cell apoptosis. AXIN2 functions as an oncogene; nevertheless, it has been revealed by multiple studies to exert oncogenic effects on colorectal and oral cancers (24, 35). It has been reported that AXIN2 positively correlates with oncogenic lncRNA FGD5-AS1 expression within pancreatic carcinoma (26). However, the correlation of AXIN2 with NK cells has not been reported. We demonstrated that AXIN2 is a miR-1275 downstream target gene via expression validation and luciferase assay. AXIN2 exhibits high expression within pancreatic cancer NK cells and is negatively modulated by miR-1275. Consistent with our previous results, the results of functional experiments also showed that AXIN2 overexpression was able to significantly attenuate the restorative effect of upregulated miR-1275 upon the killing ability of NK cells against cancer cells under hypoxic environments (36). The above results show that AXIN2 functions as a miR-1275 downstream target gene, and hypoxia can inhibit NK cell tumor-killing ability by inhibiting miR-1275/AXIN2 in turn.

However, under hypoxic conditions, there may be other regulatory pathways for AXIN2 to participate in the killing ability of NK cells. As we know, the killing effect of NK cells on tumor cells is mainly through the release of granzyme, perforin and other cytotoxic factors (37). Moreover, TCF1 restricts the expression of granzymes and AXIN2 is a target of TCF1 (38). As **Figure S1** indicated, the TCF1 protein level was notably increased in NK cells under hypoxic conditions when compared with that in normoxia conditions. Similar to our findings, Orikasa et al. reported that hypoxic conditions significant upregulation of TCF1 mRNA in human dental pulp stem cells (39). Hence, we speculated that under hypoxic conditions, TCF1 is upregulated in NK cells, affecting NK cell granzymes’ expression, thereby weakening NK cells’ killing effect on tumor cells through regulating AXIN2. This hypothesis needs to be verified by conducting more experiments in the future.

In summary, we report that the hypoxic environment inhibits miR-1275 expression and thus promotes AXIN2 expression in NK cells, resulting in impaired NK cell killing ability against pancreatic cancer cells and promoting immune escape. These findings reveal a novel mechanism underlying the dysfunction of NK cells and offer potential targets for pancreatic cancer treatment.

## Data availability statement

The datasets presented in this study can be found in online repositories. The names of the repository/repositories and accession number(s) can be found in the article/**Supplementary Material**.

## Ethics statement

The studies involving humans were approved by Xiangya Hospital Ethics Committee. The studies were conducted in accordance with the local legislation and institutional requirements. The participants provided their written informed consent to participate in this study. Ethical approval was not required for the studies on animals in accordance with the local legislation and institutional requirements because only commercially available established cell lines were used.

## Author contributions

ZO: Data curation, Funding acquisition, Validation, Writing – original draft, Writing – review & editing. YL: Formal Analysis, Funding acquisition, Methodology, Writing – review & editing. DX: Formal Analysis, Methodology, Writing – review & editing. ZL: Conceptualization, Formal Analysis, Writing – review & editing.

## Supplementary material

Supplementary Figure 1 TCF1 protein level in NK-92 cells under hypoxia or normoxia conditions. NK-92 cells were cultivated under hypoxia and normoxia for conditions 24 h and TCF1 protein level in NK-92 cells was detected by western blot assay. ***P < 0.01*, in comparison with Normoxia.
