# Supplementary figures and images for: Hypoxia mediates immune escape of pancreatic cancer cells by affecting miR-1275/AXIN2 in natural killer cells
Source: Front Immunol. 2023 Nov 15;14:1271603. doi: 10.3389/fimmu.2023.1271603 (PMC10684956; doi:10.3389/fimmu.2023.1271603)

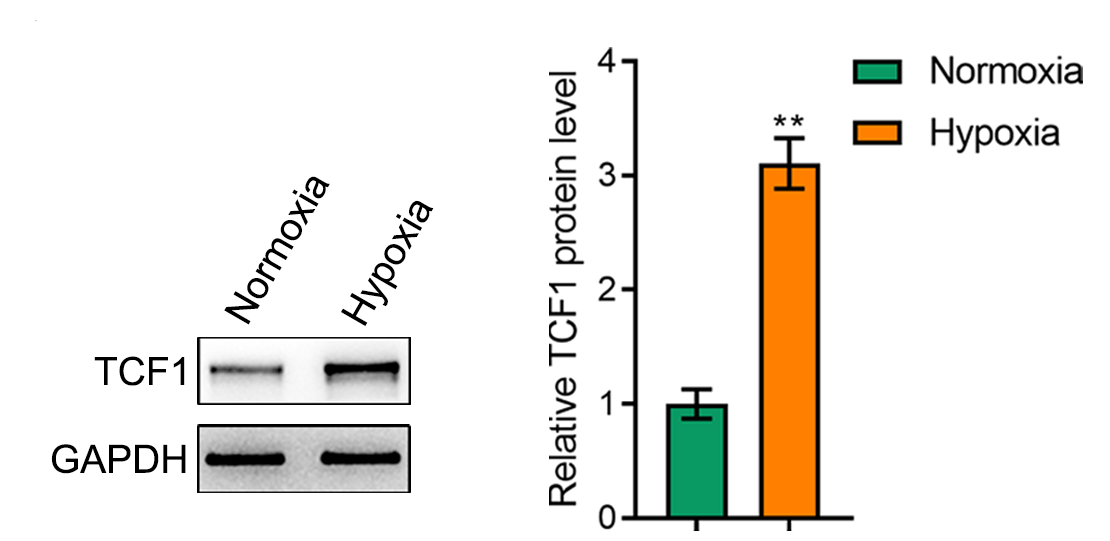

Supplement: Supplementary Figure 1 — TCF1 protein level in NK-92 cells under hypoxia or normoxia conditions. NK-92 cells were cultivated under hypoxia and normoxia for conditions 24 h and TCF1 protein level in NK-92 cells was detected by western blot assay. **P < 0.01, in comparison with Normoxia. [file Image_1.tif]
