# Supplementary material for: Hypoxia mediates immune escape of pancreatic cancer cells by affecting miR-1275/AXIN2 in natural killer cells
Source: Front Immunol. 2023 Nov 15;14:1271603. doi: 10.3389/fimmu.2023.1271603 (PMC10684956; doi:10.3389/fimmu.2023.1271603)
Supplement: Supplementary file 2 [file Table_1.docx]

Table S1 the sequence of miRNA and overexpression vector construction primer.

| Name | F（5’-3’） | R （5’-3’） |
| --- | --- | --- |
| Agomir-NC | UUCUCCGAACGUGUCACGUTT | ACGUGACACGUUCGGAGAATT |
| Agomir-1275 | GUGGGGGAGAGGCUGUC | CAGCCUCUCCCCCACUU |
| Antagomir-NC | CAGUACUUUUGUGUAGUACAA |  |
| Antagomir-1275 | GACAGCCUCUCCCCCAC |  |
| AXIN2 overexpression (pcDNA3.1) | ctagcgtttaaacttaagcttATGAGTAGCGCTATGTTGGTGACT | tgctggatatctgcagaattcTCAATCGATCCGCTCCACTT |
