# Supplementary material for: Hypoxia mediates immune escape of pancreatic cancer cells by affecting miR-1275/AXIN2 in natural killer cells
Source: Front Immunol. 2023 Nov 15;14:1271603. doi: 10.3389/fimmu.2023.1271603 (PMC10684956; doi:10.3389/fimmu.2023.1271603)
Supplement: Supplementary file 3 [file Table_2.docx]

| Name |  | Sequence（5’-3’） |
| --- | --- | --- |
| miR-1275 | Forward | RT；GTCGTATCCAGTGCGTGTCGTGGAGTCGGCAATTGCACTGGATACGACGACAGC  F:GCGGTGGGGGAGAG |
|  | Reverse | CAGTGCGTGTCGTGGA |
| U6 | Forward | CTCGCTTCGGCAGCACA |
|  | Reverse | AACGCTTCACGAATTTGCGT |
| AXIN2 | Forward | CAACACCAGGCGGAACGAA |
|  | Reverse | GCCCAATAAGGAGTGTAAGGACT |
| β-actin | Forward | TTCCAGCCTTCCTTCCTGGG |
|  | Reverse | TTGCGCTCAGGAGGAGCAAT |

Table S2 primer sequence
